# Charting Early Brain Plasticity in Radiological Training: Functional Brain Reorganization During Early Radiological Expertise Acquisition

**DOI:** 10.3390/brainsci15121279

**Published:** 2025-11-28

**Authors:** Weilu Chai, Yuxin Bai, Jia Wu, Hongmei Wang, Jimin Liang, Xuemei Xie, Chenwang Jin, Minghao Dong

**Affiliations:** 1 Engineering Research Center of Molecular and Neuro Imaging of Ministry of Education, School of Life Science and Technology, Xidian University, Xi’an 710071, China; 2 Xi’an Key Laboratory of Intelligent Sensing and Regulation of Trans-Scale Life Information, School of Life Science and Technology, Xidian University, Xi’an 710126, China; 3 Key Laboratory of Intelligent Perception and Image Understanding of Ministry of Education, School of Artificial Intelligence, Xidian University, Xi’an 710071, China; 4 School of Foreign Studies, Northwestern Polytechnical University, 127 West Youyi Road, Xi’an 710072, China; 5 Department of Medical Imaging, First Affiliated Hospital of Medical College, Xi’an Jiaotong University, Xi’an 710049, China; 6 Department of Radiology, First Affliated Hospital of Xi’an Jiaotong University, Xi’an 710061, China; 7 School of Electronic Engineering, Xidian University, Xi’an 710071, China

**Keywords:** radiological expertise, resting-state fMRI, regional homogeneity, recursive feature elimination-support vector machine, neuroplasticity

## Abstract

Background/Objectives: Radiological expertise draws on semantic knowledge and perceptual–cognitive mechanisms that support diagnostic reasoning. Early radiological training is a formative period when key cognitive processes begin to integrate. Nevertheless, how the brain pattern of early radiological expertise reorganizes during the first weeks of clinical exposure remains unknown, as prior work has relied mainly on cross-sectional designs comparing mature experts to beginners. Methods: We therefore conducted a longitudinal resting-state fMRI study in radiology interns (*n* = 43; 41 valid) scanned before and after short-term training. Behavioral performance improved significantly after training (*p* < 0.01). Regional homogeneity (ReHo) was computed for 246 Brainnetome ROIs for each subject. Results: Using a Support Vector Machine (SVM)-based recursive feature elimination (RFE) pipeline, 14 of these 246 features were identified as most discriminative, spanning regions involved in visual, semantic, memory, attentional, and decision-making processes. An SVM trained on these features effectively differentiated pre- and post-training brain states (training set: 86.67% accuracy, AUC = 0.97; validation set: 81.82% accuracy, AUC = 0.72). Conclusions: The observed neuroplastic changes provide direct evidence that multidimensional cognitive functions reorganize early in radiological expertise development and offer neural targets to inform evidence-based curriculum design, personalized training, and brain-targeted interventions (e.g., neuromodulation or neurofeedback) in radiology education.

## 1. Introduction

Beyond the accumulation of semantic medical knowledge, radiologists rely on perceptual–cognitive mechanisms to detect subtle yet critical differences within visually homogeneous pathological regions, thereby enabling accurate interpretation of medical images [1]. This perceptual skill—radiological expertise—is acquired through extensive exposure to clinical diagnostic cases [2,3] and is widely regarded as being supported by experience-dependent neuroplastic changes in the brain [4].

From a learning perspective, early stages of skill acquisition can induce measurable changes in neural processing, as short-term training—even over days or weeks—has been shown to reorganize neural responses and cortical circuits, reflecting rapid experience-dependent plasticity [5,6]. In radiology, the early stage is particularly critical, as newly graduated interns with foundational knowledge need intensive diagnostic training and broad exposure to clinical radiographs to advance quickly to independent competence. At this phase, learners are developing the ability to extract salient visual features [7,8], link them to emerging semantic and conceptual knowledge [9], form initial memory templates for recurrent patterns [10], allocate attention more selectively to diagnostically meaningful cues [11], and begin to engage basic decision-making processes under uncertainty [12]. These capacities are not yet mature or optimized; instead, they are undergoing rapid, experience-driven adjustment [13]. Thus, the earliest weeks of clinical exposure represent a window in which foundational components of radiological expertise are actively being assembled, making this period especially informative for identifying the initial neuroplastic changes that support subsequent skill development. However, most previous neuroimaging studies rely on cross-sectional comparisons between mature radiologists and novices and cannot reveal how early training-induced functional reorganization unfolds [14,15,16,17]. The initial neuroplastic patterns that mark the onset of radiological expertise development remain largely unexamined.

Due to the limited understanding of how radiological expertise emerges during early stages of training at the neural level, resting-state fMRI (rs-fMRI) provides a unique window into the brain’s intrinsic functional architecture. Free from task-related demands, rs-fMRI enables the examination of spontaneous neural activity in a task-independent context [18,19]. Resting-state activity, reflected in low-frequency spontaneous fluctuations, has been shown to encode prior experiences and support ongoing internal representations [20,21]. This makes rs-fMRI a powerful tool for investigating experience-dependent brain changes [22]. Among various rs-fMRI metrics, Regional Homogeneity (ReHo) is a reliable and reproducible index that quantifies local synchronization of spontaneous neural activity [23,24,25], making it particularly suitable for detecting subtle changes in functional organization during the early development of radiological expertise.

To address this gap in understanding how radiological expertise first emerges at the neural level, a longitudinal resting-state fMRI study was conducted in radiology interns (*n* = 43; 41 valid datasets). Using ReHo as a sensitive index of local spontaneous synchrony, we examined whether short-term clinical training (four weeks) induces early functional reorganization in intrinsic brain networks. Data-driven feature extraction and machine-learning classification (RFE–SVM) were applied to identify training-responsive neural signatures. We hypothesized that, during the early stage of radiological training, a ReHo-based brain pattern of expertise development would be detectable within networks supporting visual, semantic, memory, attentional, and decision-making processes. By directly linking behavioral gains with intrinsic functional reorganization, this study aims to characterize the earliest neuroplastic changes that initiate expertise development—an aspect overlooked in prior cross-sectional expertise research—and provide an empirical basis for optimizing evidence-based radiology training.

## 2. Materials and Methods

### 2.1. Subjects

To capture neural adaptations at the earliest stage of expertise acquisition, we recruited newly graduated radiology interns at the start of their clinical rotation—drawn from the same medical school with an identical preclinical curriculum emphasizing classroom-based imaging instruction and with no prior structured diagnostic or interpretive experience—thereby maximizing sensitivity to short-term training-induced functional changes and largely controlling prior radiological exposure at the cohort level. In total, 43 healthy, right-handed Chinese radiology interns were recruited (22 males; mean age = 22.47 ± 1.02 years). Handedness was assessed using the Edinburgh Handedness Inventory [26]. All participants had normal or corrected-to-normal visual acuity and confirmed having no smoking habits, alcohol abuse, MRI scan restrictions, or history of mental or health disorders.

### 2.2. Experimental Procedure

We used a longitudinal within-subject design. All participants completed a four-week rotation in the X-ray department (radiological training), 6 days a week. On average, they reviewed 25–35 X-ray cases each day. Particularly, they were required to identify pathologies from X-ray images, which heavily relied on visual recognition skill, and submit medical reports. Each participant had a radiology tutor providing feedback based on the interns’ report. By the end of the rotation, each intern had interpreted 600–840 cases, as recorded in the hospital’s Picture Archiving and Communication System. Participants completed prescreening questionnaires, behavioral testing, and MRI scanning before training and repeated the behavioral testing and MRI after the four-week rotation. Prescreening self-report questionnaires were administered before MRI scanning to exclude individuals with visual expertise in other domains (e.g., birds, chess, cars, aircraft, animals, plants, and mushrooms). All participants reported no such expertise. Behavioral testing was administered to quantify radiological expertise performance using the Radiological Expertise Test (RET), following our prior work [27,28]. To avoid potential task-induced effects on resting brain activity, behavioral testing was scheduled after MRI acquisition.

### 2.3. Behavioral Test

We quantified radiology expertise pre- and post-training with the RET. The same image set was used at both time points. In RET, participants viewed 100 chest radiographs and indicated (i) nodule presence/absence and (ii) a benign/malignant judgment for each case. The 100 cases were curated from the department’s teaching archive by three independent senior radiologists (each with >15 years’ experience). The set comprised three difficulty levels (approximately 50%, 30%, and 20% of the cases from easy to hard). Each image contained zero to several pulmonary nodules; cases unrelated to nodules were not included. Sixty-five images with 1–3 nodules served as positive cases, and 35 images without nodules served as negatives. In the context of the RET, interns were asked to detect whether a pulmonary nodule was present or absent and, if present, localize the suspected nodule. To ensure task feasibility for interns, all abnormal cases were screened by three experts for clear radiographic conspicuity under routine display settings. In general, nodules of approximately ≥10 mm in diameter are visually detectable under these conditions, and extremely subtle lesions below novice detectability were excluded by expert consensus rather than by a single size cutoff. Because the chest radiographs used for RET circulate internally via the hospital’s Picture Archiving and Communication System (PACS), they cannot be publicly released without formal institutional authorization.

Ambient lighting was controlled, and natural light was excluded. The entire task procedure was fully explained to each subject, and the task started after the subject correctly described the entire procedure to the experimenter. Before RET, the subjects were informed that each X-ray image might contain zero to an uncertain number of nodules. The subjects were supposed to identify all the abnormalities as well as their confidence for each round of identification within 5 s. RET was accomplished using in-house software (China Software Patent No. 2018SR036699), which records patients’ judgment on the presence or absence of nodules, confidence in each choice and the response time for each decision (note that 0 to *n* rounds of decisions were made for each image). The software automatically compared the answers given by the subjects and the ground-truth answers provided by the aforementioned senior radiologists. To control for potential practice effects, the same 100 images selected for the radiological expertise task (RET) were used with randomized order for each subject, ensuring that no image was repeatedly shown in exactly the same order for both pre-training and post-training assessments. Additionally, to minimize the possibility of bias, no test images were discussed or mentioned during the training sessions. Each subject underwent thorough practice trials before the actual assessments began, allowing them to familiarize themselves with the task format without any influence from the test images used in the actual pre- and post-training evaluations.

### 2.4. Behavioral Data Analysis

Behavioral performance on the RET before and after training was evaluated using the Jackknife Alternative Free-Response Receiver Operating Characteristic method [29], which enables population-level generalization of diagnostic performance. The resulting figure-of-merit, defined as the area under the free-response receiver operating characteristic curve, served as the score of the RET, with values ranging from 0 (poor) to 1 (perfect). We also calculated RET response time before and after training, defined as the average latency of diagnostic decisions recorded during the task. Normality of the difference scores was confirmed using the Shapiro–Wilk test (all *p* > 0.05). Accordingly, paired *t*-tests were conducted to compare RET scores and response times before and after the four-week radiological training program.

### 2.5. MRI Data Acquisition

All participants completed comprehensive physical and neurological examinations before scanning. To avoid task-induced modulation of central representations, behavioral testing was scheduled after MRI acquisition. Imaging was performed on a 3.0-Tesla MRI system (EXCITE, General Electric, Milwaukee, WI, USA.) at the First Affiliated Hospital of the Medical College, Xi’an Jiaotong University (Xi’an, China). To control for time-of-day effects [30], all sessions ran between 8:30 and 12:00 a.m. Each visit included a resting-state scan and a high-resolution structural scan. A standard birdcage head coil and foam padding were used to stabilize the head and attenuate scanner noise. Before scanning, participants were instructed to keep their eyes closed, remain still, and stay awake; immediately afterward, they were asked whether they had fallen asleep during the procedure.

Resting-state fMRI was acquired as 185 volumes, each consisting of 35 axial slices (TR/TE = 2000/30 ms; flip angle = 90°; field of view = 240 mm; matrix = 64 × 64; voxel size = 3.8 × 3.8 × 5.0 mm^3^; slice thickness = 4 mm; inter-slice gap/layer spacing = 0 mm). The corresponding acquisition time was 370 s, and the resting-state scan session lasted 8 min 20 s in total. High-resolution T1-weighted structural images were obtained with a 3D magnetization-prepared rapid gradient-echo sequence.

### 2.6. MRI Data Analysis

**Figure 1:**
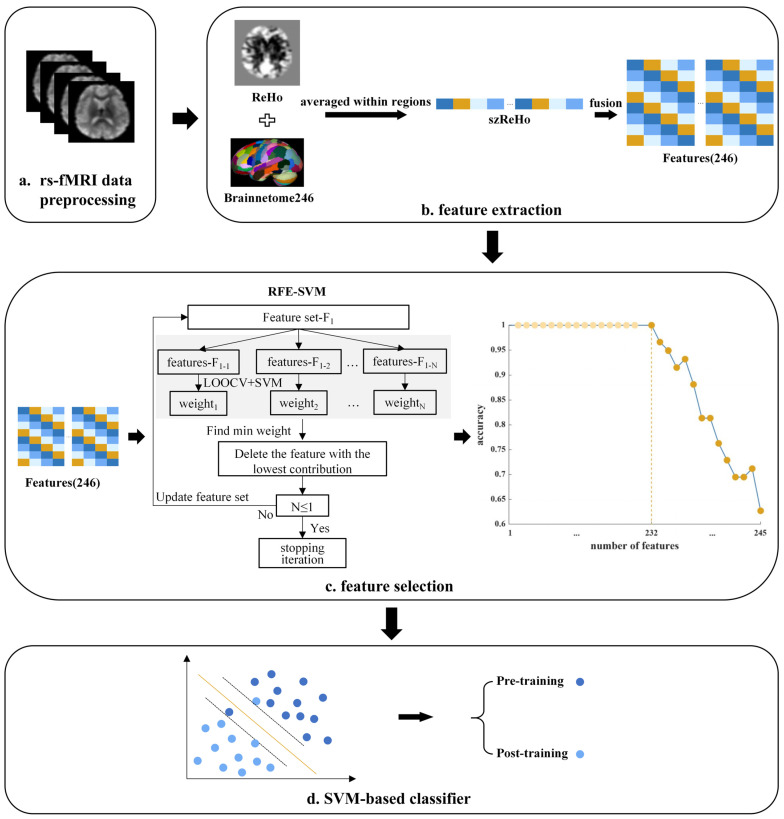
Data analysis pipeline. (**a**) Resting-state fMRI (rs-fMRI) data were preprocessed. (**b**) Feature extraction: voxel-wise ReHo maps were standardized (szReHo) and averaged within 246 ROIs defined by the Brainnetome atlas, yielding 246 regional ReHo features. (**c**) Feature selection: a recursive feature elimination–support vector machine (RFE–SVM) approach iteratively removed the least informative features until optimal classification accuracy was reached. The curve shows classification accuracy across feature subsets; the dark orange point marks the final retained feature set. (**d**) Classification: the retained features were used in a linear SVM classifier to distinguish resting-state brain patterns before and after radiological training.

As illustrated in Figure 1, for all resting-state MRI data acquired from participants before and after radiological training, we conducted preprocessing, feature extraction, and feature selection. Following this, we applied SVM classification to identify brain regions that differentiate between trained and untrained participants’ resting-state brain patterns. The specific procedure is detailed below.

#### 2.6.1. MRI Data Preprocessing

Preprocessing was performed using Statistical Parametric Mapping (SPM12) and the Data Processing Assistant for Resting-State fMRI (DPARSF 4.5) [31]. The first 10 volumes were discarded to allow magnetization equilibrium and participant adaptation. Functional images underwent slice-timing correction, realignment (motion correction) to the first image of the session, and spatial normalization to the MNI template. Nuisance regression included the 24-parameter head-motion model, white-matter and CSF mean signals, and a linear trend; global signal regression was not applied. Temporal band-pass filtering (0.01–0.08 Hz [32])—appropriate for TR = 2000 ms (sampling rate 0.5 Hz; Nyquist 0.25 Hz)—was then applied to each voxel time series to attenuate low-frequency drifts and high-frequency physiological noise. Head motion was quantified using framewise displacement (FD) as defined by Power et al. (2012) [33]; two participants were excluded due to mean FD > 0.2 mm. For the remaining 41 participants, between-session head motion did not differ significantly.

#### 2.6.2. Feature Extraction

Because our aim was to identify a distributed pattern of training-related plasticity rather than test each ROI in isolation, we did not use traditional univariate group comparisons as the primary analytic strategy. In such patterns, some ROIs may indeed show clear mean differences, but other ROIs may not reach univariate significance and yet become informative when considered jointly with the rest of the network. This logic follows classic multivariate fMRI work showing that distributed patterns can carry robust information even when single voxels/regions show weak or inconsistent univariate effects [34]. RFE–SVM leverages these joint contributions in the multivariate ReHo feature space, whereas separate *t*-tests or ANOVAs ignore inter-regional covariance.

To obtain whole-brain functional region ReHo features, our feature extraction process consisted of two steps: first, generating a voxel-wise ReHo map, and then producing a region-wise ReHo map.

##### Generation of Voxel-Wise Regional Homogeneity Map

ReHo quantifies, at the voxel level, the similarity between a voxel’s BOLD time series and those of its adjacent voxels using Kendall’s coefficient of concordance (KCC). Conceptually, ReHo indexes the local synchrony of nearby signals [25]. Resting-State fMRI Data Analysis Toolkit (REST) [35] was used to compute ReHo. For each subject, every voxel was assigned the KCC between its time series and those of its 26 nearest neighbors to generate the voxel-wise ReHo map. A brain mask derived from the MNI template (consistent with the normalization space) was applied to exclude non-brain voxels and reduce noise, and each ReHo map was standardized by dividing voxelwise values by the subject’s mean KCC within the mask. Finally, the maps were spatially smoothed with an isotropic 6 mm FWHM Gaussian kernel.

##### Generation of Region-Wise Regional Homogeneity Map

Once a voxel-wise ReHo map had been obtained from each subject, voxel-wise ReHo values were averaged within the Brainnetome atlas (246 regions of interest, ROIs) [36], yielding a region-wise ReHo map with 246 mean ReHo features (one per ROI) for each subject. This procedure localized the signal to functionally defined brain regions and reduced dimensionality (from tens of thousands of voxels to 246 regional features), thereby helping to mitigate overfitting given the modest sample size (*n* = 41). For each subject, the 246-dimensional ReHo vectors from the pre- and post-training scans were labeled as pre-training or post-training samples and then served as the input to the RFE–SVM framework, which iteratively removed less informative features to obtain a subset of ReHo features whose joint pattern best discriminated pre- from post-training resting-state brain activity. No voxel-level statistical comparisons were conducted; therefore, no cluster-level correction (e.g., FDR or GRF) was required or applied before ROI averaging.

Prior expertise research has shown that skill acquisition can alter the regional clustering of resting-state BOLD activity in task-relevant areas, consistent with the principle that neurons that work together tend to fire together [13]. Thus, we selected ReHo rather than ALFF because ReHo quantifies the local coherence of spontaneous activity, whereas ALFF mainly reflects the amplitude of low-frequency fluctuations at single voxels and does not directly index their temporal coordination with surrounding tissue.

#### 2.6.3. Feature Selection

Feature selection is crucial in MRI data analysis, as it effectively reduces dimensionality, eliminates irrelevant data, enhances the accuracy of the classification model, and improves the interpretability of results [37]. To eliminate irrelevant brain ROIs and thereby improve the interpretability of brain networks potentially involved in visual expertise, the 246 ReHo feature vectors input into the classifier needed to be reduced through feature selection. An RFE-SVM approach was employed, which iteratively removes features contributing the least to classification accuracy until further elimination decreases accuracy [38]. The specific steps were as follows:

1. The resulting beta maps were standardized to a 0–1 range using mean–variance normalization across all ReHo features.
2. RFE was then used to assess feature importance via the classifier and progressively eliminate features contributing the least to classification accuracy, reducing feature dimensionality. Our implementation of RFE followed these steps:
  a. Input all ReHo feature data and class labels as training samples, train the SVM classifier, and evaluate the model’s classification accuracy using leave-one-out cross-validation (LOOCV);
  b. Sequentially extract the feature weights from the SVM model, rank the features based on the absolute values of their weights, and determine their contribution to classification accuracy;
  c. Remove the feature with the smallest weight (i.e., the feature ranked last), representing the feature with the least contribution to classification accuracy; and
  d. Continue iterating until any additional feature removal leads to a drop in classification accuracy.

As a result of the feature selection process, we retained key features, represented by the dark orange dots in Figure 1. We then used an SVM classifier to assess whether these features exhibited high discriminative ability, where high discriminative ability was defined as the capacity to distinguish between resting-state brain patterns before and after radiological training.

#### 2.6.4. Support Vector Machine Classifier

The SVM is a margin-based method from statistical learning theory, originally formulated for binary classification tasks [39]. SVM learns a maximum-margin separating hyperplane; with a kernel, this boundary is estimated in an implicit feature space without explicit mapping. For neuroimaging applications, linear SVMs are commonly preferred because their decision function is easier to interpret [40]. Accordingly, we employed a linear soft-margin SVM optimized with the hinge loss. The classifier was implemented using the LIBSVM toolbox [41].

#### 2.6.5. Training the Classifier and Evaluating Its Performance

To evaluate whether the features retained after feature selection can effectively distinguish between resting-state brain activity before and after radiological training, we trained an SVM model using these features. Among the 41 valid samples, 30 were randomly designated as the training dataset, used to train the SVM model, with cross-validation applied to assess its classification performance. The remaining 11 samples, serving as the validation dataset and not involved in the training process, were used for extrapolation testing, also using cross-validation, on the trained SVM model. All references to LOOCV in this manuscript denote leave-one-subject-out cross-validation, both during feature selection (RFE) and during training/validation of the SVM classifier. It is important to note that the classification threshold was not optimized with any additional steps beyond the LOOCV used for model evaluation. The threshold was determined based on cross-validation performance, balancing accuracy and sensitivity across the training samples. No further threshold optimization steps were performed.

According to the LOOCV results, the model’s performance metrics—including accuracy, sensitivity, and specificity—were calculated as follows:

(1) $$Accuracy=\frac{TP+TN}{TP+FN+TN+FP}$$

(2) $$Sensitivity=\frac{TP}{TP+FN}$$

(3) $$Specificity=\frac{TN}{TN+FP}$$

where TP, FN, TN, and FP denote, respectively: trained participants predicted as trained (true positives), trained participants predicted as untrained (false negatives), untrained participants predicted as untrained (true negatives), and untrained participants predicted as trained (false positives). We also summarized threshold-independent performance using the area under the ROC curve (AUC); higher AUC values (closer to 1) indicate better discrimination by the SVM.

### 2.7. Correlation Analysis

To investigate the relationship between changes in the homogeneity of retained brain regions and behavioral development in radiological expertise, we conducted correlation analyses between changes in ReHo values of these brain regions and RET scores before and after training. As normality assumptions were not satisfied for part of the ReHo change variables, Spearman correlation was employed. Multiple comparisons were controlled using the Benjamini–Hochberg false discovery rate (FDR) correction, with significance set at q < 0.05.

## 3. Results

### 3.1. Results of Behavior Data Analysis

**Figure 2:**
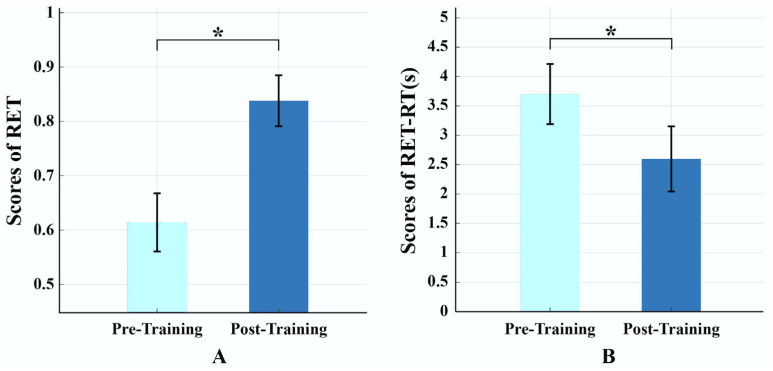
Results of behavioral tests pre- and post-training. (**A**) Significant increase in radiological expertise test (RET) scores, indicating enhanced diagnostic accuracy. (**B**) Significant reduction in response time during RET, reflecting faster decision-making. Note: * indicates the significant differences *p* < 0.05; light blue denotes pre-training, and dark blue denotes post-training.

During the four-week radiological training period, each participant reviewed 600–840 cases. As shown in Figure 2, behavioral performance improved significantly after training. RET scores increased from 0.614 ± 0.053 (pre-training) to 0.838 ± 0.047 (post-training), t(42) = 24.98, *p* < 0.001, Cohen’s d = 4.45; RET response time decreased from 3.71 ± 0.63 s (pre-training) to 2.53 ± 0.34 s (post-training), t(42) = –13.40, *p* < 0.001, Cohen’s d = –2.34. These findings indicate that radiological training significantly enhanced behavioral performance in radiological expertise.

### 3.2. Results of Mri Data Feature Selection

**Figure 3:**
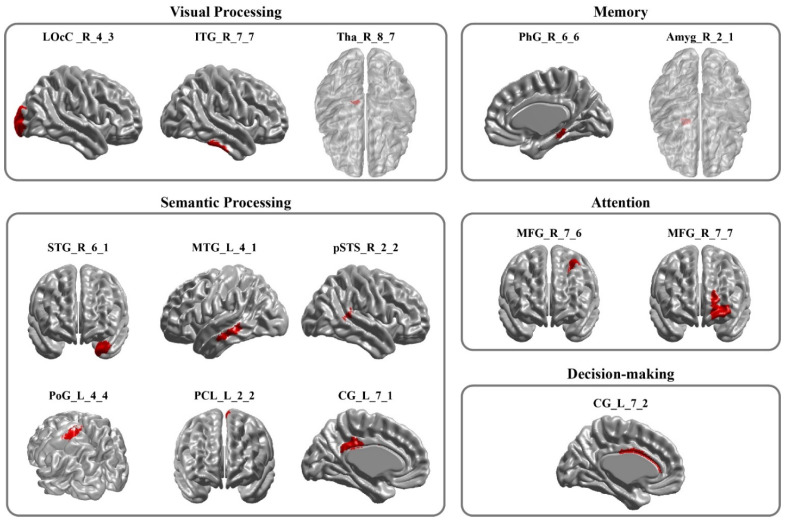
The Retained Brain Regions. This figure highlights the brain regions that contribute to classification in distinguishing pre- and post-radiological training resting-state brain activity. Brain regions identified by Regional Homogeneity (ReHo)–based feature selection and Recursive Feature Elimination–Support Vector Machine (RFE-SVM) that discriminated pre- from post-training resting-state activity. Regions are grouped by associated cognitive domains: visual processing, memory, semantic processing, attention, and decision-making. Abbreviations: LOcC, lateral occipital cortex; ITG, inferior temporal gyrus; Tha, thalamus; PhG, parahippocampal gyrus; Amyg, amygdala; STG, superior temporal gyrus; MTG, middle temporal gyrus; pSTS, posterior superior temporal sulcus; PoG, postcentral gyrus; PCL, paracentral lobule; CG, cingulate gyrus; MFG, middle frontal gyrus.

After feature selection, 14 features remained, representing discriminative brain regions, defined by their ability to distinguish between resting-state brain patterns before and after radiological training. As shown in Figure 3, these 14 features correspond to the following brain regions—right thalamus (Tha, Tha_R_8_7), right lateral occipital cortex (LOcC, LOcC_R_4_3), right inferior temporal gyrus (ITG, ITG_R_7_7), right parahippocampal gyrus (PhG, PhG_R_6_6), right amygdala (Amyg, Amyg_R_2_1), right middle frontal gyrus (MFG, MFG_R_7_6 and MFG_R_7_7), right superior temporal gyrus (STG, STG_R_6_1), right posterior superior temporal sulcus (pSTS, pSTS_R_2_2), and left middle temporal gyrus (MTG, MTG_L_4_1), left paracentral lobule (PCL, PCL_L_2_2), left postcentral gyrus (PoG, PoG_L_4_4), left cingulate gyrus (CG; posterior CG: CG_L_7_1, anterior CG: CG_L_7_2). For transparency, we report pre- and post-training mean ReHo values (±SD) of the 14 selected regions in Table 1, but statistical inference centers on this multivariate pattern.

### 3.3. Results of Classifier Performance

**Figure 4:**
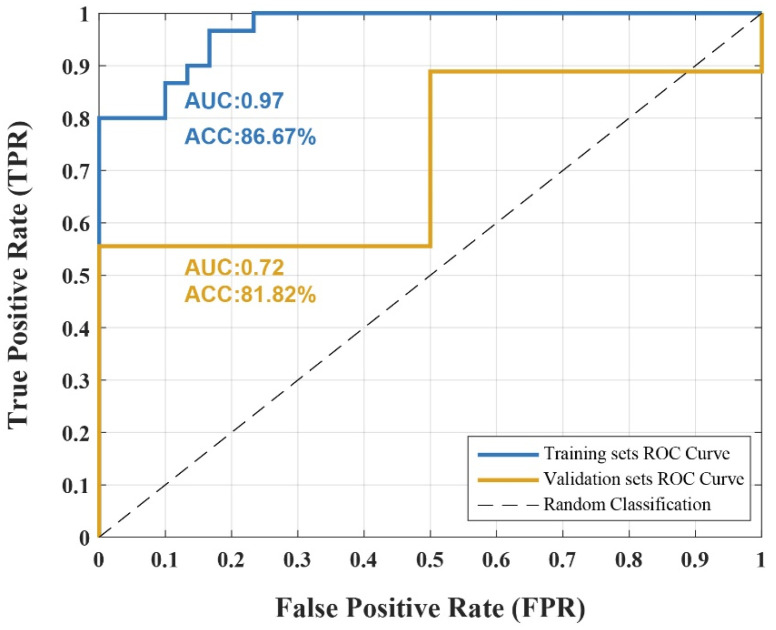
Classification Performance of SVM Model. Receiver operating characteristic (ROC) curves of the support vector machine (SVM) classifier trained on the retained features identified. The model achieved an accuracy (ACC) of 86.67% with an area under the curve (AUC) of 0.97 in the training set, and an ACC of 81.82% with an AUC of 0.72 in the independent validation set. The dashed line indicates chance-level classification.

To determine if the retained features can reliably differentiate between resting-state brain patterns before and after radiological training, we applied these features to train an SVM model. The SVM classifier achieved an accuracy of 86.67% on the training dataset, with specificity and sensitivity values of 86.67% and 66.67%, respectively. In the validation dataset, the classifier attained an accuracy of 81.82%, with specificity and sensitivity values of 50% and 88.89%, respectively. As shown in Figure 4, the AUC values for the training and validation datasets were 0.97 and 0.72, respectively. Additionally, a permutation test (1000 iterations) was conducted to confirm that classification accuracy exceeded chance level (*p* < 0.01), further supporting model robustness. These results demonstrate that the retained features, representing distinct brain regions, had high discriminative power, effectively distinguishing between pre- and post-training resting-state brain patterns.

### 3.4. Results of Correlation Between Behavioral and Mri Data Changes

The results showed that changes in the ReHo values of two semantic-related brain regions within the retained brain regions were significantly associated with changes in RET scores.

**Figure 5:**
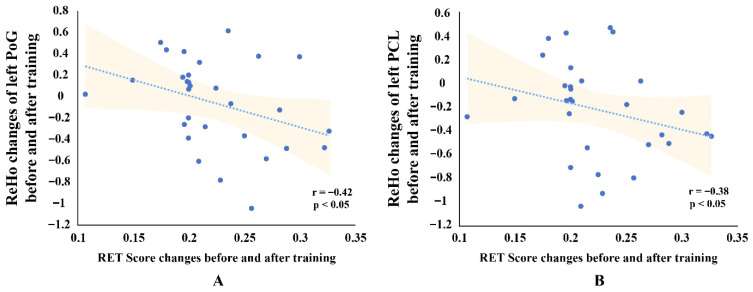
Scatterplots Showing Correlations Between ReHo Changes and RET Scores for two brain regions. (**A**) Correlation between Radiological Expertise Test (RET) score changes and Regional Homogeneity (ReHo) changes in the left postcentral gyrus (PoG). (**B**) Correlation between RET score changes and ReHo changes in the left paracentral lobule (PCL). Each scatterplot shows the linear trend line with 95% confidence intervals (shaded areas). Spearman correlation coefficients (r) and significance levels (*p* < 0.05, FDR-corrected) are displayed in the lower right corner.

As shown in Figure 5, changes in ReHo values in the left PoG and left PCL were significantly negatively correlated with changes in RET scores after FDR correction (left PoG: *r* = −0.42, *p* < 0.05; left PCL: *r* = −0.38, *p* < 0.05). These findings suggest that changes in homogeneity in the left PoG and left PCL at least exhibit an inverse trend with behavioral performance in radiological expertise, with decreased homogeneity in these regions showing a tendency to be associated with enhanced performance.

## 4. Discussion

This study shows that brief radiology training is accompanied by intrinsic functional reorganization that parallels behavioral gains in diagnostic accuracy. Using a multivariate ReHo–SVM framework, we identified a distributed neural signature that reliably distinguishes pre- from post-training states, spanning visual, semantic, memory, attentional, and decision-making networks. These findings show that four-week-training-induced neural adaptations are already detectable at rest during the early stage of expertise formation.

### 4.1. Neural Networks Supporting Visual Processing in Radiological Training

In radiological image interpretation, radiologists’ visual processing begins with a rapid, global assessment of the image (holistic processing) before progressing to a detailed examination of a limited number of critical low-level visual feature details [8]. Our findings identified three regions—the right Tha, right LOcC, and right ITG—as central to early development of radiological expertise (Figure 3). These regions match findings from mature expert–novice contrasts—such as ITG in radiology [15], LOcC in car expertise [42], thalamus in chess expertise [43], and occipito-temporal cortex (LOcC/ITG) in music reading [44]—whereas our results show that reorganization in these visual nodes is already evident at the early stage of expertise development.

In visual processing, visual information flows from the retina to the lateral geniculate nucleus (LGN) in the Tha, progresses through the early visual cortex (V1/V2) for low-level feature extraction, and reaches higher-order regions like the ITG for high-level feature integration [45,46]. Specifically, the LGN within the Tha transmits visual inputs from the retina to the early visual cortex (V1/V2) [47]. The LOcC (referred to as the occipital polar cortex [36]), part of the early visual cortex (V1/V2) [48], processes low-level features such as line segments [49], orientation [50], and luminance contrast [51] before relaying this information to higher-order areas. The ITG, as a high-level processing region, integrates these low-level features into holistic visual representations [52,53].

Our findings demonstrate that radiological training was associated with neuroplastic changes in the resting-state homogeneity of key visual processing regions. Specifically, in our study, ReHo values decreased in the right Tha and right LOcC, while increasing in the right ITG, reflecting distinct adaptations in regions supporting different stages of visual processing (Table 1). This pattern, characterized by reduced coherence in regions involved in visual input and low-level processing alongside enhanced coherence in the ITG, suggests an improved ability to integrate visual input features into holistic perceptions, thereby reducing the cognitive load associated with low-level visual feature details input and processing during image recognition. These adaptations likely form the neurophysiological basis for the observed improvements in interns’ radiological expertise, bringing their processing patterns closer to those of radiologists with higher recognition accuracy—characterized by stronger holistic processing and reduced reliance on extensive low-level visual feature details [7,8].

In summary, these findings provide novel insights into the neural mechanisms underlying the visual processing aspects of radiological expertise development. Intensive four-week X-ray training not only improved behavioral performance (Figure 2) but also drove neuroplastic changes in the resting-state homogeneity of the Tha, LOcC, and ITG. We propose that these neural adaptations may have improved holistic perception integration, enhanced the efficiency of visual input transmission and low-level feature processing, and potentially aligned interns’ visual perceptual patterns more closely with those of experienced radiologists.

### 4.2. Neural Networks Supporting Semantic Processing in Radiological Training

In X-ray diagnosis, radiologists rely on their expertise to integrate visual perceptual information with prior verbal information, such as patient demographics, medical history, symptoms, and lab results. This visual-verbal information integration enables experts to surpass novices in diagnostic accuracy by processing visual perceptual cues into semantic knowledge through integrating them with diagnostic verbal information, thereby forming more comprehensive semantic interpretations [54,55]. Our findings identified brain regions associated with this expertise semantic process, including the left MTG, right STG, right pSTS, left PCL, left PoG, and left posterior CG (Figure 3). These regions match findings from mature expert–novice contrasts—such as left MTG in music-reading and Scrabble expertise [44,56], right pSTS/STG in radiology [17], left PoG in music-reading expertise [44], and posterior cingulate/precuneus within car and chess expertise [42,57]—whereas our results show that reorganization in these semantic nodes is already evident at the early stage of expertise development.

In real-world visual recognition, semantic processing, gained through real-world perceptual experiences, encodes and stores semantic knowledge from object features (like color, sound, and motion) into accessible semantic representations [9]. This processing extends beyond mere visual perceptual feature extraction [58], integrating conceptual information from both visual and verbal attributes [59]. The brain regions identified in our study play distinct roles in this semantic processing that integrates verbal and visual perceptual features. The STG, for example, encodes semantic features from verbal input [60], while the MTG and STS integrate these verbal features with visual information processed by the ITG to form cohesive semantic concepts [58]. Additionally, the PCL and PoG are implicated in cross-modal integration of visual and verbal features [61,62], while the posterior CG bridges semantic and memory networks [9,63].

In our study, post-training results revealed decreased ReHo values in the left MTG, right STG, right pSTS, left PCL, left PoG, and left posterior CG, suggesting a functional reorganization of semantic processing networks (Table 1). This decreased coherence functional reorganization may reflect a shift toward more specialized and efficient processing of diverse diagnostic features, including both visual and verbal elements, enabling radiology interns to explore a broader range of diagnostic possibilities and adopt a more comprehensive approach. Notably, the decreased ReHo values in the left PCL and left PoG showed a trend toward correlation with the improved behavioral performance of interns following their radiological training, further supporting this possibility (Figure 5).

In summary, our findings suggest that radiological training enhances the semantic processing abilities of radiology interns, enabling them to integrate visual perceptual and verbal features more effectively. Changes in resting-state homogeneity in the identified brain regions reflect the neuroplastic adaptations supporting expertise development. Specifically, the decreased ReHo values in the semantic processing regions indicate a transition from novice-like reliance on simplified processing of limited features to a more differentiated approach involving a wider range of diagnostic scenarios. Such functional reorganization provides a physiological mechanism that facilitates the interns’ improved diagnostic performance after training, as evidenced by the observed enhancements in radiological task performance (Figure 2).

### 4.3. Neural Networks Supporting Memory in Radiological Training

Radiologists rely on their accumulated correct diagnostic experiences, stored in long-term memory, to retrieve and apply radiological perceptual expertise. Our findings identified the right PhG and right Amyg as significant regions in the early development of radiological expertise (Figure 3). Consistent with findings in mature visual experts from domains such as chess [43,64], Scrabble [56], and bird and car recognition [65], we further show that the PhG and Amyg exhibit early training-linked functional reorganization, suggesting that mnemonic scaffolds begin adapting at the onset of expertise formation.

In visual expertise memory processing, chunking theory suggests experts outperform novices by encoding domain-specific knowledge into multiple “chunks” stored in long-term memory [66]. Template theory, extending chunking, posits these chunks serve as templates, allowing experts to populate them with variable short-term memory information, thus aiding retrieval [10]. In memory processing, the PhG, like the hippocampus, is vital for long-term memory [67]. The PhG supports the encoding of visual recognition chunks into long-term memory [68] and is crucial for associative memory during visual recognition, aiding chunk retrieval [69]. Additionally, the Amyg, through emotional regulation, enhances the storage and retrieval of long-term memory chunks in the PhG [70,71].

In our study, increased ReHo values in the right PhG and right Amyg after training suggest enhanced neural coherence in these regions, likely supporting the encoding and retrieval of radiological expertise chunks (Table 1). Positive emotional associations with correct diagnoses during training may have further reinforced these processes, as evidenced by plasticity changes in the resting-state activity of these regions. This enhanced storage and retrieval capacity likely contributed to the improved diagnostic accuracy observed in behavioral assessments.

In summary, following radiological training, we speculate that interns’ correct diagnostic experiences in chest X-ray cases, combined with positive emotional responses, were encoded as radiological expertise chunks in long-term memory, supported by plasticity changes in the right PhG and right Amyg. During post-training diagnostic tasks, these chunks were retrieved and populated with variable short-term memory information from current images, enabling more accurate recognition. This training-induced enhancement in long-term memory encoding and retrieval provides a neurophysiological basis for the improved diagnostic accuracy observed post-training, as evidenced by improved behavioral outcomes (Figure 2).

### 4.4. Neural Networks Supporting Attention in Radiological Training

Radiological expertise is characterized by enhanced attention to critical visual features, facilitated by the interplay between bottom-up processing of salient new stimuli and top-down guidance based on perceptual experience [72]. Our results highlight the pivotal role of the right MFG in this process (Figure 3). Prior mature expert–novice studies have implicated the MFG in attentional control across radiology [15], cars [42], and chess [64]; while our data reveal that attentional hubs already reorganize during the early phase of radiological training.

Attention is the cognitive process by which goal-driven signals (top-down) interact with sensory information (bottom-up) to selectively focus on relevant objects [11]. The MFG serves as a hub between the dorsal and ventral attention networks, mediating shifts in attention through the integration of goal-driven signals and sensory inputs [73]. Specifically, the MFG transmits top-down goal-driven signals from the dorsal attention network to guide the ventral network in focusing on key visual stimuli, while also relaying bottom-up signals from salient new stimuli in the ventral network back to the dorsal network in response to unexpected stimuli [11]. Post-training, we observed decreased average ReHo values in the MFG, suggesting functional segregation (Table 1). This may reflect a refinement in attentional control, allowing interns to process X-ray images more efficiently with reduced neural resource allocation. This result suggests that radiological training induces neurophysiological plasticity in the MFG, enhancing attentional control efficiency and underpinning the interns’ improved performance.

In summary, we propose that during the four-week radiological training, interns’ repeated exposure to critical visual stimuli facilitated visual expertise formation, actively guided by their developing expertise to identify target stimuli. This likely induced plastic changes in the right MFG’s resting-state homogeneity, enhancing its role in attention shifting and supporting radiological expertise development, as evidenced by improved behavioral outcomes (Figure 2).

### 4.5. Neural Networks Supporting Decision-Making in Radiological Training

Decision-making is a cornerstone of radiological expertise, particularly when resolving perceptual conflicts such as ambiguous or overlapping image patterns. Radiologists with advanced expertise demonstrate efficient decision-making, characterized by rapid and accurate judgments [74]. Our findings underscore the importance of the left anterior cingulate gyrus (CG) in this process (Figure 3). Building on evidence that mature experts show plasticity in decision-making regions such as the anterior CG in radiology [17] and other expertise domains (e.g., music reading [44] and Scrabble [56]), we find that intrinsic synchrony in this same region is already reshaped after only four weeks of early clinical exposure—indicating that decision-making networks begin adapting from the very onset of expertise development.

Decision-making involves selecting actions by evaluating potential outcomes and refining conflicting choices through experience [12,75]. The anterior CG is crucial in decision-making, continuously adjusting strategies and resolving conflicts based on outcomes to facilitate optimal action selection [76,77]. Post-training, we observed decreased average ReHo values in the anterior CG, indicative of functional specialization (Table 1). This adaptation likely reflects enhanced decision-making efficiency and conflict resolution during X-ray interpretation, achieved with fewer coherent neural resources. This result suggests that radiological training fosters neural plasticity in the anterior CG, potentially enhancing decision-making efficiency and contributing to expertise development.

In summary, we propose that interns refined their decision-making strategies during radiological training through tutor feedback outcomes, improving conflict resolution and inducing plastic changes in the left anterior CG’s resting-state homogeneity. This suggests that the training experience induced neuroplastic changes in the anterior CG, which served as a physiological basis for more efficient decision-making in conflicting diagnoses of visual lesions, thereby supporting the development of radiological expertise and leading to improved diagnostic outcomes (Figure 2).

### 4.6. An Integrated Account of Early Radiological Expertise

Taken together, our results indicate a coordinated, early-stage reconfiguration across five systems that jointly support radiological interpretation. In the visual system (Tha, LOcC, ITG), the pattern is consistent with a shift from emphasis on low-level input toward higher-level integration. In the semantic system (MTG, STG, pSTS, PCL, PoG, posterior cingulate), the pattern suggests more differentiated, context-dependent mappings between percepts and diagnostic knowledge. Memory hubs (PhG, Amyg) show changes consistent with rapid scaffolding and retrieval of long-term expertise templates. Attentional control (right MFG) aligns with leaner, more selective allocation of resources, and decision-making circuitry (anterior CG) aligns with more efficient conflict monitoring and resolution. Collectively, these adaptations imply that perception, semantic integration, memory retrieval, attentional selection, and decision arbitration become more streamlined after short training, in parallel with behavioral gains.

### 4.7. Future Directions: Brain-Based Targeting to Enhance Radiology Training

Overall, this study demonstrates that a short-term training intervention can drive specific neurophysiological adaptations that translate into measurable cognitive-behavioral gains. By delineating the brain regions whose resting-state homogeneity reliably tracks early expertise, we provide a concrete set of neural targets for future intervention studies—such as real-time fMRI neurofeedback, transcranial magnetic stimulation, or transcranial direct-current stimulation—designed to accelerate learning. These findings therefore link environment, brain plasticity, and behavior in a closed loop that can be leveraged to personalize training protocols and optimize radiology curricula. The trained SVM model developed in this study serves as a powerful tool to distinguish between pre- and post-training resting-state brain activity by integrating brain feature data, such as ReHo. This method allows us to track and quantify neuroplastic changes associated with early radiological expertise, offering insights into how specific brain regions evolve during training. While this model is primarily applied within the context of radiology training, the classification-based feature selection approach it employs, combined with brain imaging data, has far-reaching potential in various fields. This method can be extended to domains such as sports expertise, musical proficiency, and language learning, where brain plasticity plays a critical role in skill acquisition. By leveraging neuroimaging data to track neural adaptations, this approach provides an effective means to evaluate and optimize training interventions across these diverse fields. Additionally, the identification of key ReHo features not only enhances the model’s ability to differentiate expertise levels but also provides a set of neural targets that can be used to guide future neurofeedback or brain stimulation interventions such as TMS and tDCS. These neural targets offer a means to personalize training and interventions, making them more precise and effective in enhancing learning and performance in expertise-driven domains.

### 4.8. Limitations

This study has several limitations. First, the sample size of 41 participants is modest; it was appropriate for the exploratory nature of this study. We employed LOOCV to limit overfitting. While the 86.7%/81.8% train–validation accuracies support reasonable generalization, the lower validation AUC and specificity = 0.50 point to residual generalization decline, plausibly due to the small sample. Future work with larger cohorts is warranted to strengthen generalizability. Second, the four-week training window limits depiction of the full trajectory of radiology-training-related plasticity, and future research needs to conduct longer longitudinal follow-up to chart neural changes as interns progress toward mature-expert profiles. Third, although the same 100 images were randomized at pre- and post-training to mitigate practice effects, test–retest learning cannot be fully ruled out, and future research needs to include a no-training control arm with novel post-test images to better isolate training-specific effects. Fourth, we did not explicitly and systematically quantify individual prior exposure to radiological interpretation; future studies will incorporate standardized indices of pre-rotation exposure to further minimize residual variance. Finally, because ReHo indexes local synchrony, decreases in ReHo reflect reduced intra-regional coherence; our interpretation that such reductions may align with functional specialization or efficiency is not unique, and the underlying mechanism remains ambiguous. Future research needs convergent measures to disambiguate these possibilities.

## 5. Conclusions

This study shows that even four weeks of radiology training is accompanied by intrinsic functional reorganization that parallels measurable gains in diagnostic performance. Using ReHo with a machine-learning classifier, we identified a distributed neural signature across visual, semantic, memory, attentional, and decision networks that reliably differentiates pre- from post-training states, indicating early experience-dependent plasticity. Practically, these neural markers provide actionable targets to personalize training and motivate the exploration of brain-targeted supports (e.g., neurofeedback, TMS/tDCS) to further enhance radiology education. Together, the results offer a neurobiological basis for evidence-informed optimization of intern radiology training.

## Figures and Tables

**Table 1:** Mean ReHo values (±SD) of the brain regions with high discriminative power before and after radiological training.

Functions	Brain Regions		Pre Training (Mean ± SD)	Post Training (Mean ± SD)	Cohen’s d
**Visual processing**	right ITG	ITG_R_7_7	−0.55 ± 0.48	−0.40 ± 0.39	0.34
	right LOcC	LOcC _R_4_3	0.55 ± 0.37	0.39 ± 0.40	−0.42
	right Tha	Tha_R_8_7	−0.61 ± 0.34	−0.76 ± 0.31	−0.46
**Semantic processing**	left MTG	MTG_L_4_1	0.43 ± 0.21	0.34 ± 0.20	−0.44
	right STG	STG_R_6_1	−0.61 ± 0.17	−0.65 ± 0.23	−0.20
	right pSTS	pSTS_R_2_2	0.28 ± 0.24	0.23 ± 0.32	−0.18
	left PCL	PCL_L_2_2	0.25 ± 0.38	0.24 ± 0.32	−0.03
	left PoG	PoG_L_4_4	0.65 ± 0.32	0.51 ± 0.32	−0.44
	left posterior CG	CG_L_7_1	0.80 ± 0.29	0.65 ± 0.32	−0.49
**Memory**	right PhG	PhG_R_6_6	−0.63 ± 0.37	−0.42 ± 0.30	0.62
	right Amyg	Amyg_R_2_1	−0.87 ± 0.23	−0.78 ± 0.23	0.39
**Attention**	right MFG	MFG_R_7_6	0.49 ± 0.31	0.39 ± 0.28	−0.34
		MFG_R_7_7	0.26 ± 0.38	0.12 ± 0.37	−0.37
**Decision-making**	left anterior CG	CG_L_7_2	−0.25 ± 0.35	−0.34 ± 0.33	−0.26

Abbreviations: ITG, inferior temporal gyrus; LOcC, lateral occipital cortex; Tha, thalamus; MTG, middle temporal gyrus; STG, superior temporal gyrus; pSTS, posterior superior temporal sulcus; PCL, paracentral lobule; PoG, postcentral gyrus; CG, cingulate gyrus; PhG, parahippocampal gyrus; Amyg, amygdala; MFG, middle frontal gyrus.

## Data Availability

The data that support the findings of this study are available from the corresponding author, upon reasonable request. According to the informed consent signed by participants, the study data can only be accessed by the experimenters of this project and are not intended for public release. Furthermore, the cross-border sharing or uploading of citizens’ biological data to overseas platforms requires prior written authorization from our supervisory authorities; such authorization is not currently in place.
